# *GaMYB85*, an R2R3 MYB gene, in transgenic *Arabidopsis* plays an important role in drought tolerance

**DOI:** 10.1186/s12870-017-1078-3

**Published:** 2017-08-22

**Authors:** Hamama Islam Butt, Zhaoen Yang, Qian Gong, Eryong Chen, Xioaqian Wang, Ge Zhao, Xiaoyang Ge, Xueyan Zhang, Fuguang Li

**Affiliations:** State Key Laboratory of Cotton Biology, Institute of Cotton Research of Chinese Academy of Agricultural Science (ICR, CAAS), Anyang, 455000 China

**Keywords:** MYB transcription factor, Abiotic stress tolerance, Abscisic acid

## Abstract

**Background:**

MYB transcription factors (TFs) are one of the largest families of TFs in higher plants and are involved in diverse biological, functional, and structural processes. Previously, very few functional validation studies on R2R3 MYB have been conducted in cotton in response to abiotic stresses. In the current study, *GaMYB85,* a cotton R2R3 MYB TF, was ectopically expressed in *Arabidopsis thaliana* (Col-0) and was functionally characterized by overexpression in transgenic plants.

**Results:**

The *in-silico* analysis of *GaMYB85* shows the presence of a SANT domain with a conserved R2R3 MYB imperfect repeat. The GaMYB85 protein has a 257-amino acid sequence, a molecular weight of 24.91 kD, and an isoelectric point of 5.58. *Arabidopsis* plants overexpressing *GaMYB85* exhibited a higher seed germination rate in response to mannitol and salt stress, and higher drought avoidance efficiency than wild-type plants upon water deprivation. These plants had notably higher levels of free proline and chlorophyll with subsequent lower water loss rates and higher relative water content. Germination of *GaMYB85* transgenics was more sensitive to abscisic acid (ABA) and extremely liable to ABA-induced inhibition of primary root elongation. Moreover, when subjected to treatment with different concentrations of ABA, transgenic plants with ectopically expressed *GaMYB85* showed reduced stomatal density, with greater stomatal size and lower stomatal opening rates than those in wild-type plants. Ectopic expression of *GaMYB85* led to enhanced transcript levels of stress-related marker genes such as *RD22, ADH1, RD29A, P5CS,* and *ABI5.*

**Conclusions:**

Our results indicate previously unknown roles of *GaMYB85,* showing that it confers good drought, salt, and freezing tolerance, most probably via an ABA-induced pathway. These findings can potentially be exploited to develop improved abiotic stress tolerance in cotton plants.

**Electronic supplementary material:**

The online version of this article (doi:10.1186/s12870-017-1078-3) contains supplementary material, which is available to authorized users.

## Background

Environmental factors, particularly drought stress, severely limit the production and distribution of many important agronomic crops worldwide [1]. In plants, different types of transcription factors (TFs), such as bZIP, NAC, AP2, WRKY, and MYB, control plant biological processes by modulating the initiation rate of target genes with the combined activation of a DNA binding domain, a nuclear localization signal, a transcription activation domain, and oligomerization sites in response to biotic or abiotic stresses [2]. MYB TFs were first recognized as oncogenes in animals; however, it has subsequently been found that MYB genes occur more widely in plants than in animals and fungi [3, 4]. Following the discovery of the first plant MYB gene in maize (*Zea mays*) [5], large numbers of MYB proteins, particularly R2R3 MYBs, have been extensively identified and characterized in different plant species, including *Arabidopsis*, apple, grape, maize, petunia, and snapdragon [6]. Furthermore, R2R3 MYBs are known to control abundant plant biological processes, such as hormone signal transduction, organ development, cell cycle progression, cellular morphogenesis, secondary metabolism, and stress responses [7, 8]. More recently, genome-wide studies of *Gossypium raimondii* have revealed 205 putative R2R3 MYB genes, which is a greater number than reported in any other dicot or monocot [9].

Structurally, MYB proteins have a highly conserved MYB or DBD (DNA-binding domain), located at the N terminus, which is well conserved across all eukaryotes, whereas the diverse C termini act as a *trans-*acting domain (TAD) that regulates a broad range of functions in MYB proteins [10, 11]. Moreover, each MYB repeat contains 52 amino acid residues with regularly spaced triplet tryptophan residues, which form a hydrophobic core structure. The MYB repeat structure is composed of three α-helices. Two helices form the HTH (helix-turn-helix) structure and contribute to the binding of target genes to the promoter region, whereas the third helix participates in DNA recognition [12, 13]. On the basis of their DNA-binding domain sequential repeats, MYB proteins are grouped into four types; MYB1-R, R2R3-MYB, R1R2R3-MYB (MYB3R), and 4R–MYB [14]. However, to improve drought tolerance in the relevant crops, mining of new MYB R2R3 TFs by functional genomics and comparative genomics studies is a promising strategy, as it is the largest family of MYB proteins of higher plants and well known to be involved in specific diverse biological, functional, and structural processes.

The phytohormone abscisic acid (ABA) has numerous functions, such as in seed germination inhibition and dormancy maintenance, stomatal regulation, flowering time, and adaptations to drought, cold, and salt stresses [15]. ABA is known to induce dehydration-responsive genes, and thus on encountering drought stress, elevated levels of ABA stimulate *cis-*acting and *trans*-acting factors, which in turn up-regulate the ABA-induced expression of MYB, NAC, WRKY, and bZIP TF genes [16–19], thereby contributing to the relief of stress conditions [20]. Many ABA-inducible genes contain a *cis*-element (ABRE; ACGTGG/TC), whereas dehydration and low temperature stress-inducible genes contain a different *cis*-element (DRE; TACCGACAT). Both these elements play important roles in stress management via ABA-dependent and -independent signal transduction cascades [15, 21]. Moreover, *MYC/MYB* recognition sequences are a pre-requisite for transcript regulation of *RD22 and ADH1,* which is induced by high ABA levels in *Arabidopsis* under drought stress [22]. In addition, different R2R3 MYB genes (*AtMYB44, AtMYB15, AtMYB60, AtMYB96, AtMYB61,* and *GbMYB5*) have been shown to be involved in stomatal regulation via ABA in response to dehydration [23–25]. R2R3 MYB TFs are implicated in diverse plant responses to abiotic stress conditions. For example, the AtMYB102 protein is reported to be an important component that integrates wounding, osmotic stress, and ABA transduction pathways in transgenic *Arabidopsis* [26]. Similarly, overexpression studies on R2R3 *MdSIMB1* [27], *OsMYB2* [28], *GmMYBJ1* [29], *GmMYBJ2* [30], *GbMYB5* [25], *GmMYB76* [31] and *SbMYB8* [32], have shown improved drought, salt, and cold tolerance in transgenic plants.

Cotton is an important textile fiber and oil seed crop [33]; however, the repercussions of climate change, particularly drought stress, are severely limiting its production globally [34]. Our laboratory previously released *Gossypium arboreum* sequencing data [35] and RNA-seq studies have revealed hormone crosstalk, whereby MYB TFs are implicated in modifying plant responses toward drought and NaCl stresses in different tissues [36]. Thus, in view of the current scenario, we ectopically expressed a novel *G. arboreum* R2R3 MYB gene, designated *GaMYB85*, in *Arabidopsis* to study its role under drought, salt, and freezing stress conditions. Collectively, our results suggest that the *GaMYB85* gene confers good drought tolerance in overexpressing transgenic *Arabidopsis* plants, which can potentially be employed in future cotton crop improvement.

## Results

### *GaMYB85* characterization and phylogenetic analysis

**Fig. 1:**
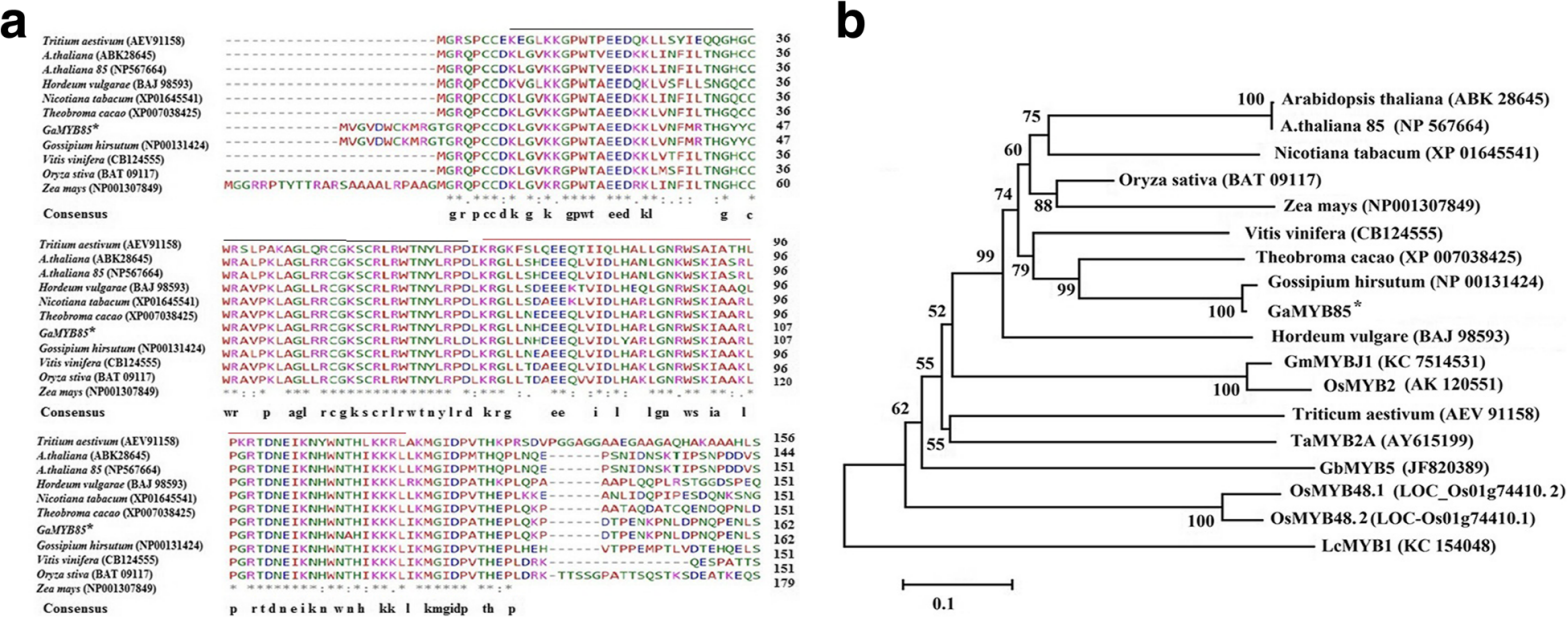
The Phylogenetic analysis and predicted structure of R2R3 MYB *GaMYB85*. **a** Homologous sequences retrieved by Blast p were aligned with *GaMYB85* showing R2 and R3 repeats represented by black and red lines respectively*.* **b** The NJ tree analysis of *GaMYB85* proteins with homologous and known R2R3 MYB monocots and dicots sequences, along with scale bar that shows the calculated distance by multiple sequence alignment (MSA). The MSA of the respective sequences were provided in Additional file 1

Previously, our mRNA-seq studies of *G. arboreum* showed multiple hormone crosstalk and tissue-selective signaling; moreover, MYB transcription factors are implicated in modifying cotton plant responses toward PEG and NaCl stresses in leaf, stem, and root [36]. Therefore, characterization of a novel gene, *GaMYB85*, was performed in transgenic *Arabidopsis* plants subjected to drought and salt stresses. The full-length CDS of *GaMYB85* is 771 bp long, and codes for a protein containing 257 amino acids with an expected molecular weight of 24.91 kD and a theoretical isoelectric point of 5.58 (http://web.expasy.org). Performing SMART analysis (available online at http://smart.embl-heidelberg.de/), the deduced 257-residue polypeptide was determined to contain a SANT domain between amino acids 38 and 86. The secondary structure of GaMYB85 includes 19.53% alpha helix, 19.53% extending chain, and 60.94% random coil sequences. *GaMYB85* alignment results between cDNA and genomic sequence retrieved from the cotton genome database revealed that the gene contains no introns (Additional file 1), and is located on chromosome 13 with a cotton ID number of cotton_A_21601. The deduced GaMYB85 protein shares a high amino acid sequence homology with AtMYB85 (At4g22680). Overexpression of this protein in *Arabidopsis* resulted in the ectopic deposition of lignin in epidermal and cortical cells of the stem [37], and it was thus designated as GaMYB85 in further studies. Multiple sequence alignment revealed high similarity between GaMYB85 and retrieved R2R3 protein homologs of various dicots and monocots, including *Gossypium hirsutum* (98%), *Nicotiana tabacum* (82%), *Arabidopsis thaliana* (79%), *Theobroma cacao* (78%), *Zea mays* (84%), *Hordeum vulgare* (80%), *Oryza sativa* (70%), *Triticum aestivum* (66%), and *Vitis vinifera* (61%), which are annotated as predicted, putative, and hypothetical, and thus their functions are still unknown. However, they show the presence of conserved R2 and R3 domains in the MYB gene (http://www.ebi.ac.uk/Tools/msa/muscle/) (Fig. 1a). Moreover, in the phylogenetic tree we constructed, we also included some R2R3 MYB genes with known functions, which confer enhanced tolerance to multiple abiotic stresses. *GaMYB85* shared high similarity and clustered together with its homologs from *G. hirsutum*, *V. vinifera,* and *T. cacao,* which indicates that they might have originated from the same common ancestor. Therefore, these results suggest that *GaMYB85* is a novel gene that confers enhanced drought tolerance (Fig. 1b). *LcMYB1*, which has a single conservered SANT domain, belongs to a MYB-related protein, and was used as an out-group in the tree.

### Overexpression of *35S:GaMYB85* in plants confers ABA hypersensitivity for seed germination and root elongation

**Fig. 2:**
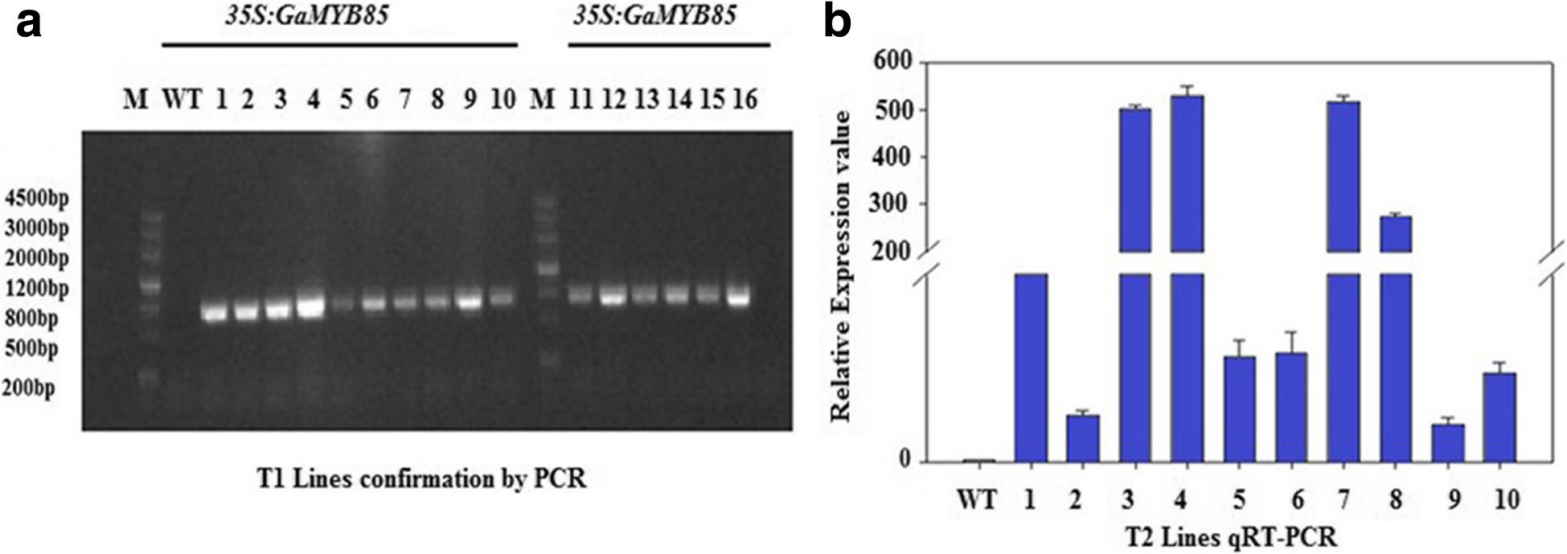
Gel and qRT-PCR analysis of *GaMYB85* transgenic plants **a** The confirmation of *GaMYB85* gene CDS (771 bp) integration in T_0_ generated overexpressed *Arabidopsis* plants. The DNA of WT (Col-0) was used as negative control (lane 1) and *35S:GaMYB85* DNA was used in (lanes 1–10, 11–16). M; DNA marker III, 1 kb. **b** Relative expression of *GaMYB85* gene in T_2_ transgenic lines by qRT-PCR having segregating ratio of 3:1 on the selective medium, three cDNA preparations were used and error bars represented with SD value. *AtUBQ*10 gene (*Accession no:* AT4G05320) was used as an internal standard in qRT-PCR

In order to gain an understanding of the possible roles of *GaMYB85* during germination and post-germination stages in response to abiotic stresses, this gene was ectopically expressed in *Arabidopsis thaliana* (Col-0) plants using the floral dip method. T_2_ seeds were selected on BASTA agar plates, and subsequently the surviving to dead plant ratio was determined (Additional files 2 and 3). Ten segregating lines with a correct segregation ratio of 3:1 were selected and transcript patterns were subsequently monitored by qRT-PCR (Fig. 2b and Additional file 5). Three lines with high gene transcript levels were selected and screened on selective media until we obtained homozygous lines. The T_3_ transgenic lines L3, L4, and L7 were selected for further analysis.

**Fig. 3:**
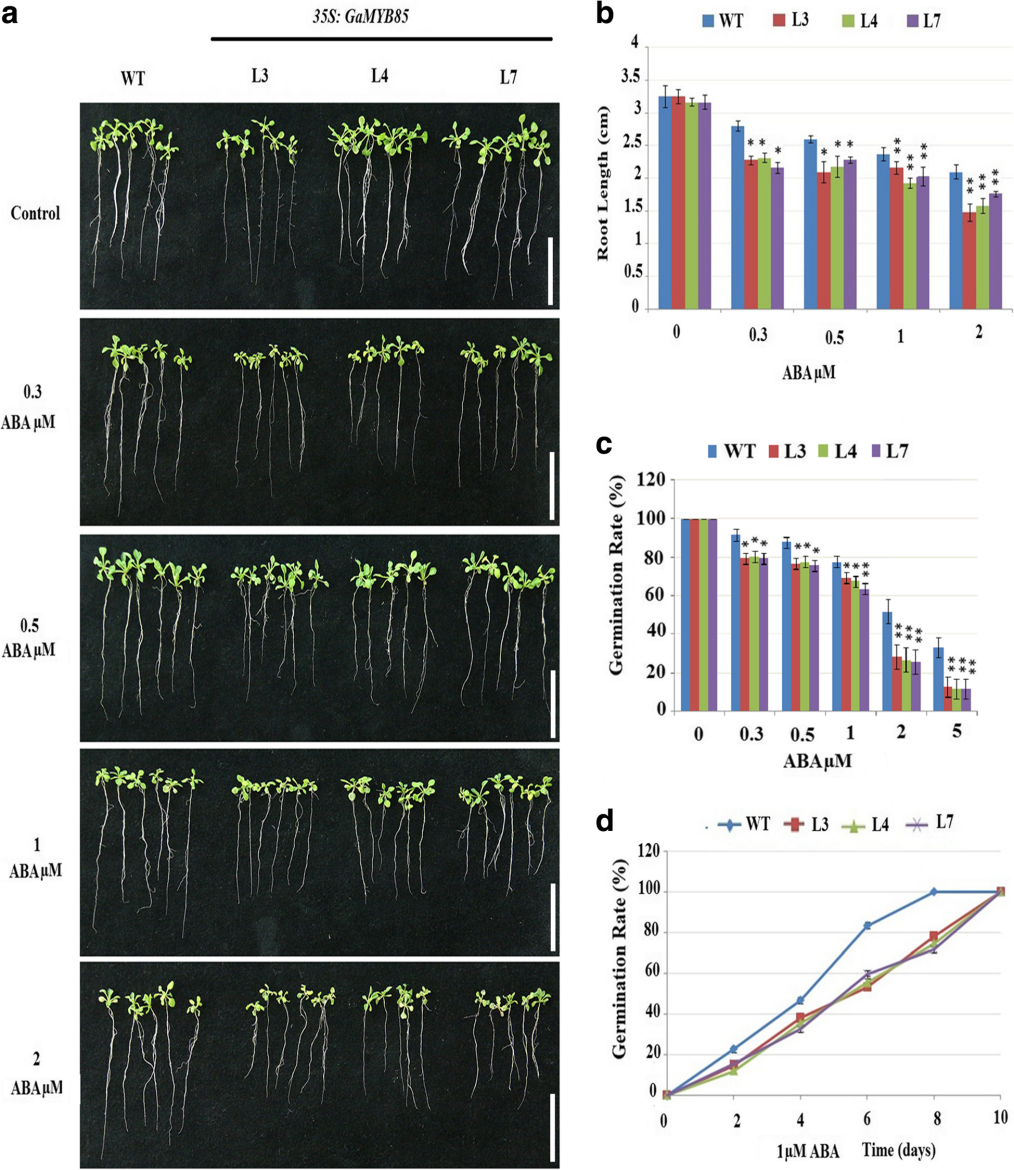
Overexpressing of *GaMYB85* modulates hypersensitivity to ABA-elicited root inhibition and seed germination rate. **a** *35S:GaMYB85* plants and WT root elongation comparisons on MS with and without varied ABA conc. (μM). The seedlings were scored and photographed after 7 days. Assay was run in triplicates, Bar line = 1 cm. **b** Quantitative comparisons of root elongation assay on ABA MS with (0, 0.3, 0.5, 1 and 2 μM). The three replicates used with 30 seedlings each, * *P* < 0.05 of mean value represented by ± SE. **c** *35S:GaMYB85* plants and WT seeds germination rates with and without ABA, the results were scored at 10th day by using 50 seeds each, ± SE (*n* = 3). **d** ABA 1(μM) supplemented plates used for time course analysis, the error bars represent SE of 3 replicates

To examine the ABA sensitivity of transgenic plants containing ectopically expressed *35S:GaMYB85*, the seeds of L3, L4, and L7 lines and WT plants were grown directly on medium containing different concentrations of ABA (0, 0.3, 0.5, 1, 2, and 5 μM), and the germination rates of these were compared. In a primary root length elongation assay, the *35S:GaMYB85* lines were more susceptible to ABA (Fig. 3a and b) than was the WT, which indicates that ABA might be involved in the root development process, and also strengthens speculation that *35S:GaMYB85* is regulated in an ABA-dependent manner. The presence of exogenous ABA had a more pronounced effect on the germination rates of *35S:GaMYB85* lines (L3, L4, and L7) than on that of the WT (Fig. 3c). Only 25% of the seeds of the *35S:GaMYB85* lines (L3, L4, and L7) were able to germinate on 2 μM ABA MS, as against 50% germination for the WT. Furthermore, a detailed time course experiment was conducted on 1 μM ABA (Fig. 3d), in which the seeds of *35S:GaMYB85* lines showed more delayed germination rates than the WT, starting from the 4th to 8th day after the onset of germination.

### Overexpression of *35S:GaMYB85* in plants confers enhanced salt and osmotic tolerance

**Fig. 4:**
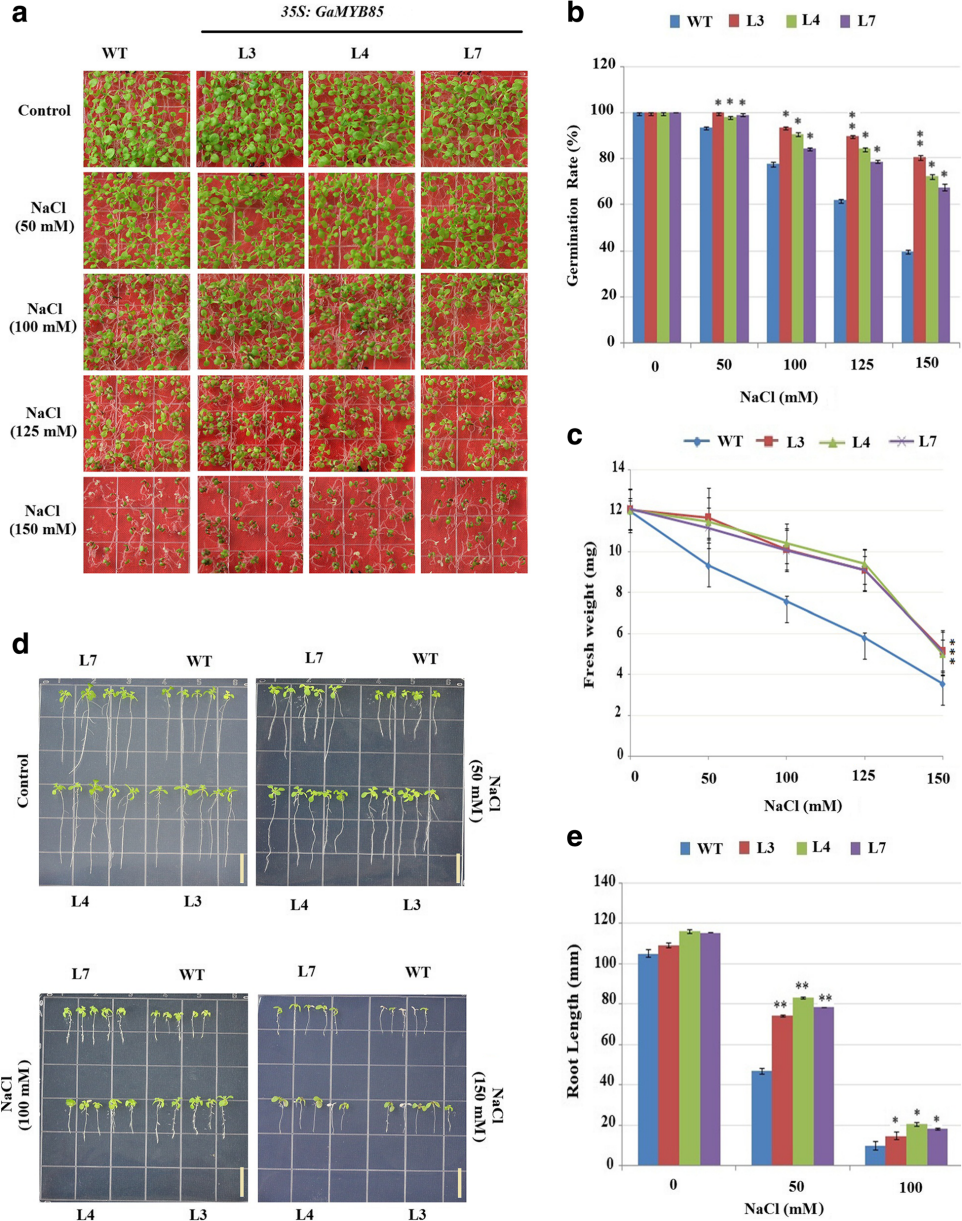
Overexpressing of *GaMYB85* enhances germination and root length in response to NaCl stress. **a** *35S:GaMYB85* plants and WT seed germination rates on MS and MS supplemented with 0-150 mM salt were photographed at 10th day of seed germination. **b** The seed germination rates plot scored at 10th day for both transgenic and WT on salt medium, 50 seeds each were used in three biological replicates (*n = 3*), *P < 0.05 calculated by t-test with means values ± SE. **c** *35S:GaMYB85* and WT seedlings fresh weights, grown on MS media with 0-150 mM NaCl, scored at 10th day of germination. 10 seedlings each used with three technical repeats and error bars with mean values ±SE. **d** Root elongation comparisons of *35S:GaMYB85* and WT on 0-150 mM NaCl MS for 6 days. The data scored from three independent growth assays. Line bar: 1 cm. **e** Quantitative comparison of root elongation of *35S:GaMYB85* and WT seedlings on MS supplemented with 0-150 mM NaCl. 30 seedlings each, (*P < 0.05) calculated by t-test and mean value ± SE (*n* = 3)

**Fig. 5:**
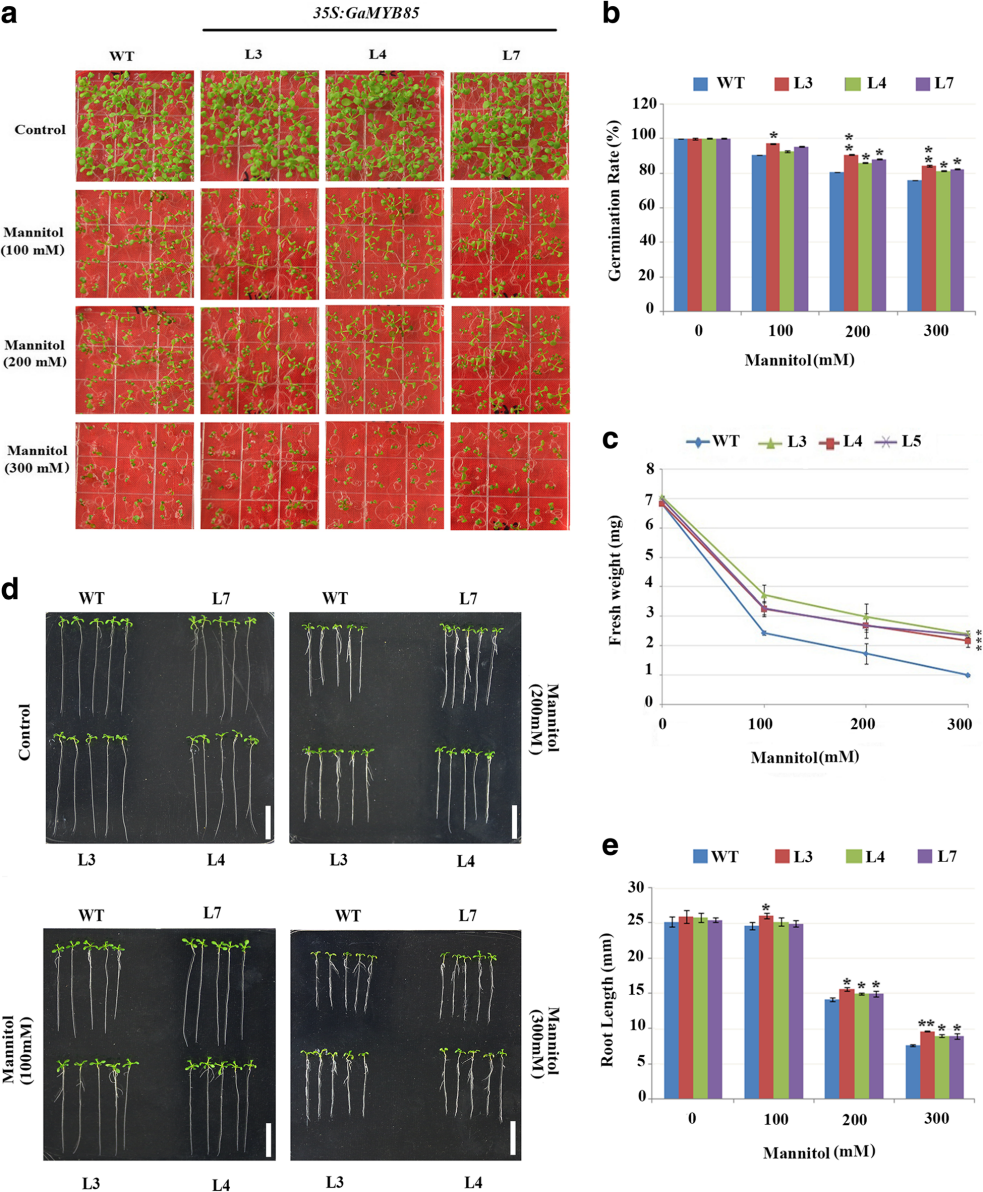
Plants overexpressing *GaMYB85* perform well in response to mannitol stress **a** *35S:GaMYB85* and WT seed germination performance on MS supplemented with 0, 100, 200, and 300 mM mannitol. Germination rates were scored on the 10th day. **b** A plot of the germination rates scored on the 10th day for *35S:GaMYB85* and WT seeds germinated on the different mannitol media. Data are the means ± SE for three replicates of 50 seeds each (*P < 0.05). **c** Fresh weights of seedlings grown on 0–300 mM mannitol MS medium scored on the 10th day. Each treatment with 10 seedlings was performed in triplicate. Data are the means ± SE (*P < 0.05). **d** Comparison of root elongation of 5-day-old seedlings of *35S:GaMYB85* and WT plants transferred to 0, 100, 200, and 300 mM mannitol MS for 6 days. Values represent the data from three independent growth assays. Scale bar: 1 cm. **e** Quantitative comparison of root elongation of *35S:GaMYB85* and WT seedlings on MS supplemented with 0–300 mM mannitol (30 seedlings each). Data are the means ± SE (*n* = 3). *P* < 0.05 determine by the t-test

For further clarification of the effects of NaCl stress on *35S:GaMYB85* plants, seed germination and post-germination growth were monitored. The results revealed 100% germination rates for control and test seeds that were grown in control MS medium. In contrast, the germination rates of *35S:GaMYB85* lines (L3, L4, and L7) were significantly higher than those of the WT on NaCl medium (50, 100, 125, and 150 mM) in a dose-dependent manner. Moreover, these plants had higher fresh weights than WT plants, which turned yellow and produced a proportion of unviable seeds (Fig. 4a, b, and c). Primary root growth is an important indicator of plant tolerance to various stress responses, and *35S:GaMYB85* transgenic seedlings showed longer roots and had greener broader rosettes than did the control when raised on 100 mM NaCl medium (Fig. 4d and e). Furthermore, compared with the WT, the germination rate and fresh weight of *35S:GaMYB85*-expressing plants were significantly higher, and growth performance was improved under 300 mM mannitol stress for 10 d (Fig. 5a, b, and c). Moreover, a root length assay on mannitol also revealed a significant difference in transgenic line root length growth on 200 and 300 mM mannitol medium from that in the WT (Fig. 5d and e). These significantly higher germination rates on simulated salt and mannitol stressed medium indicate that ectopic expression of *GaMYB85* in *Arabidopsis* confers enhanced salt and osmotic tolerance during pre- and post-seed germination stages.

### Plants with *GaMYB85* overexpression show enhanced tolerance under drought and freezing stress

**Fig. 6:**
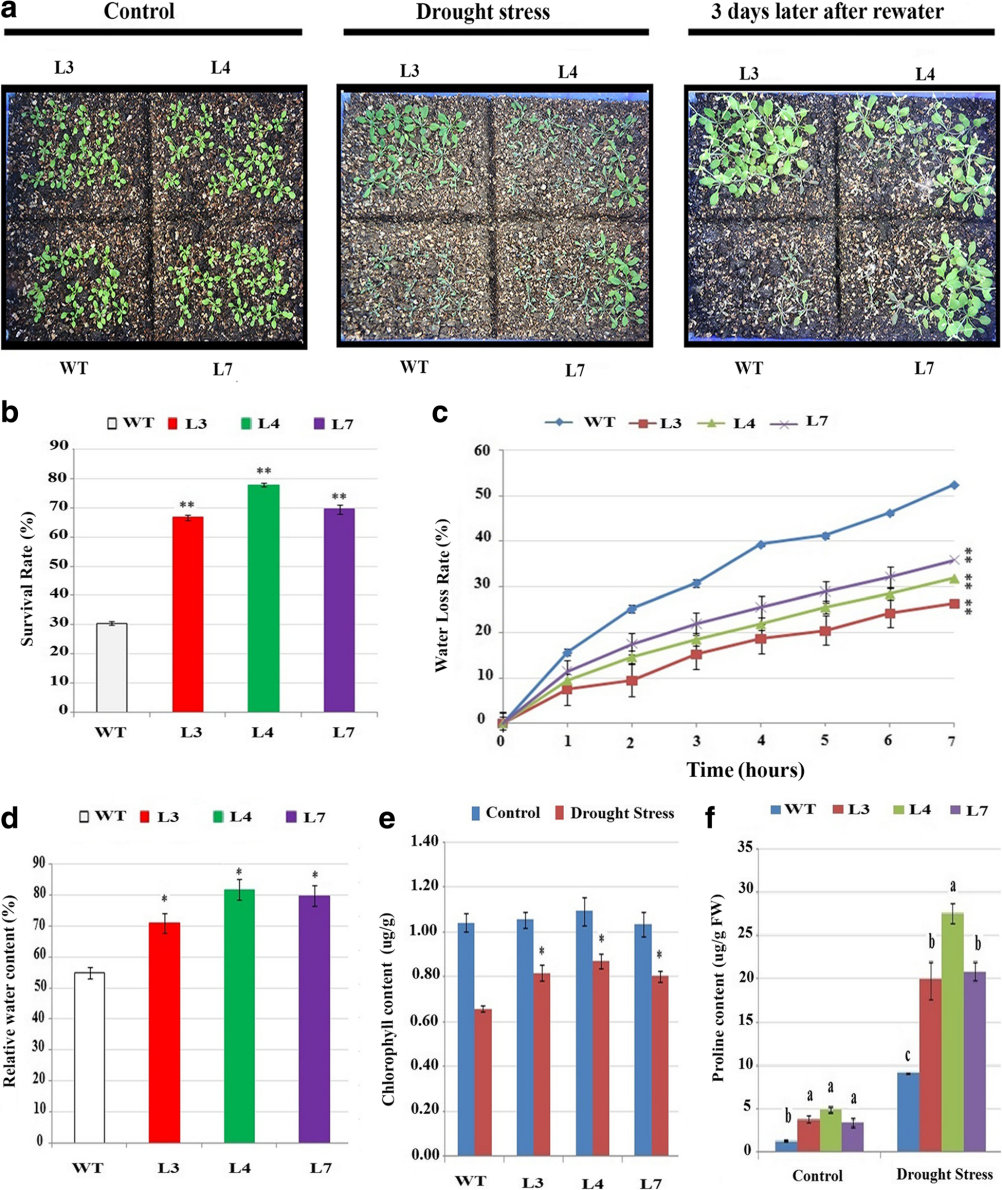
The characterization of *35S:GaMYB85* under drought stress. **a** The phenotypes of *35S:GaMYB85* and WT at the initial and late stages of dehydration and 3 days after re-watering **b** Survival percentages are the mean values ± SE of three separate assays (*n = 18*)*.* *P < 0.05 and **P < 0.01 determined by the t test. **c** Water loss rates of detached leaves of 3-week-old *35S:GaMYB85* and WT plants, expressed as percentages of initial fresh weight ± SE (*n = 10*) and (*P < 0.05). **d** Relative water content in detached leaves of 4-week-old *35S:GaMYB85* and WT plants. Data are the means ± SD (*n* = 10). Significant differences (*P < 0.05, **P < 0.01 and ***P < 0.001) were determined by Student’s t-test. **e** Proline content of *35S:GaMYB85* and WT plants under normal conditions and after 14 days of water deprivation. For concentration determinations, absorbances were measured spectrophotometrically and Duncan’s multiple range test was used for comparing means. Data are biological replicate means ± SE (*n* = 3)

**Fig. 7:**
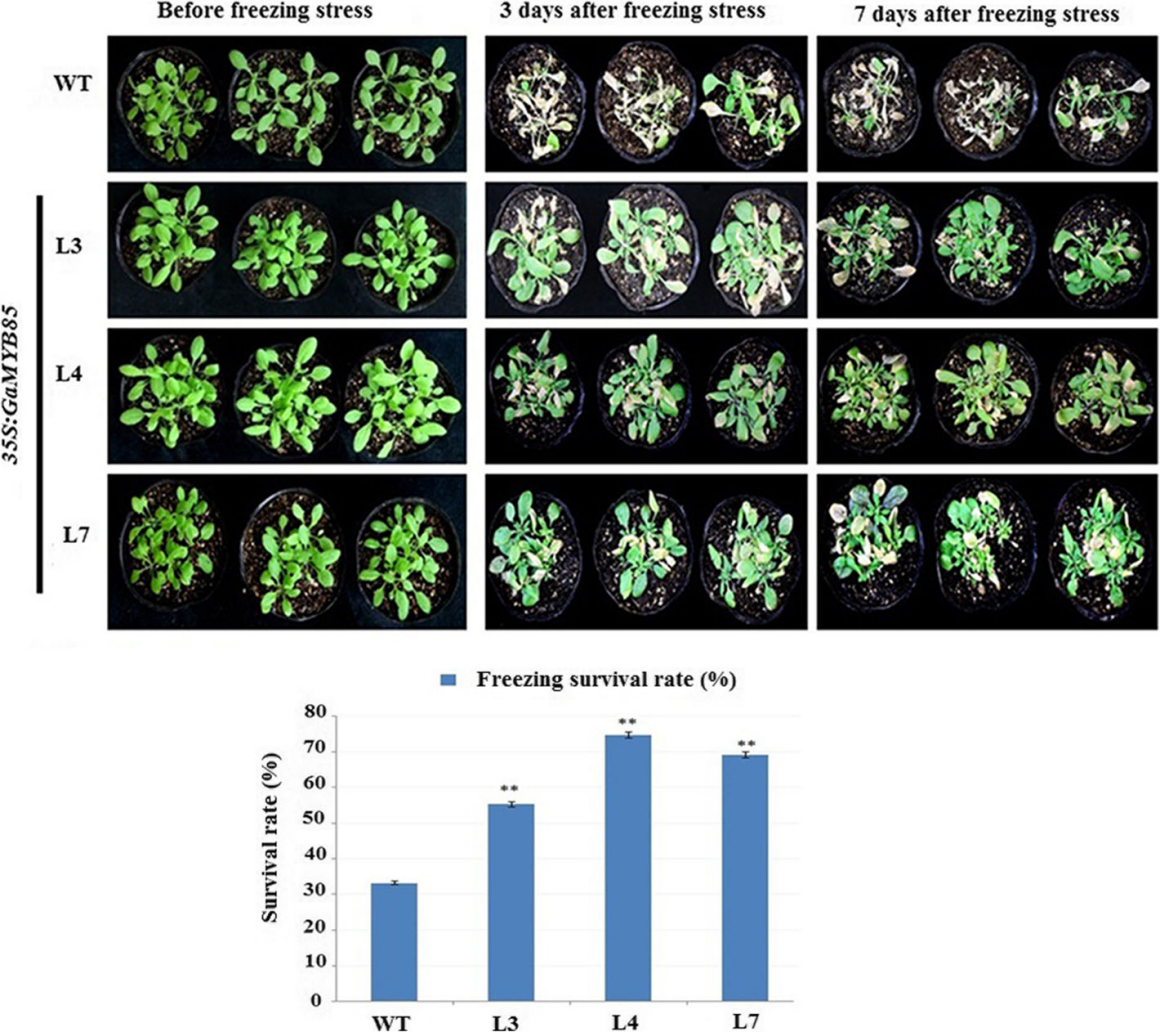
Freezing stress responses of *35S:GaMYB85* and WT plants. *35S:GaMYB85* and WT plants at −10 °C treatment, the photographs were taken after 7 days of plants revival. The survival rate percentage evaluated from three separate assays, mean values ± SD (*n = 18*) and significant difference *P < 0.05, calculated by t-test

MYB TFs have been implicated in diverse plant responses to abiotic stress conditions [26]. For a drought assay, 7-d-old transgenic and WT seedlings were transplanted to well-hydrated soil, and then 2 weeks later, grown L3, L4, and L7 plants were subjected to drought stress. The WT plants showed lower recovery rates of 35% along with severe drought symptoms, such as leaf rolling, shrinkage, and delayed growth, when compared with the 66.66%, 77.8%, and 70% recovery rates of *GaMYB85* transgenic plants (L3, L4, and L7, respectively) following re-watering for 3 d (Fig. 6a and b). In accordance with these results, the water loss rate, which is an important indicator of drought stress evaluation, was significantly lower for transgenic lines when excised leaf weights were monitored over a 0- to 7-h time interval (Fig. 6c). The relative water content (RWC) in detached leaves of L3, L4, and L7 was approximately 71%, 81.9%, and 79.9%, respectively, whereas the RWC for WT decreased by up to 55% (Fig. 6d). Subsequently, proline and chlorophyll content were evaluated in non-stressed and drought-stressed samples. The free proline content in *GaMYB85-*expressing plants was higher under both normal and drought stress conditions. The proline content in overexpressing L3, L4, and L7 plants was significantly higher (19.8, 27.5, and 20.7 μg/g, respectively) than that in the WT (almost 9 μg/g) under drought stress (Fig. 6e and f). In addition, the total chlorophyll content under the non-stressed condition was nearly the same in WT and overexpressing L3, L4, and L7 plants. However, under drought stress, the total chlorophyll content of *GaMYB85* plants was higher than that in WT plants. When 3-week-old *Arabidopsis* lines (L3, L4, and L7) ectopically expressing *GaMYB85* and WT plants were exposed to a − 10 °C treatment for 3 h, and subsequently revived in a 22 ± 1 °C growth room, the plants ectopically expressing *GaMYB85* showed improved recovery rates of 55.55%, 75%, and 69.44%, respectively. The adverse effect of freezing stress was more pronounced in the WT plants, which showed only 34% survival after 1 week of growth in the 22 ± 1 °C growth room (Fig. 7a and b). Overall, these results indicate that ectopically expressed *GaMYB85* in *Arabidopsis* plays an important role under drought and freezing stresses.

### Reduced stomatal density, increased stomatal response, and increased stomatal size in *GaMYB85* plants

**Fig. 8:**
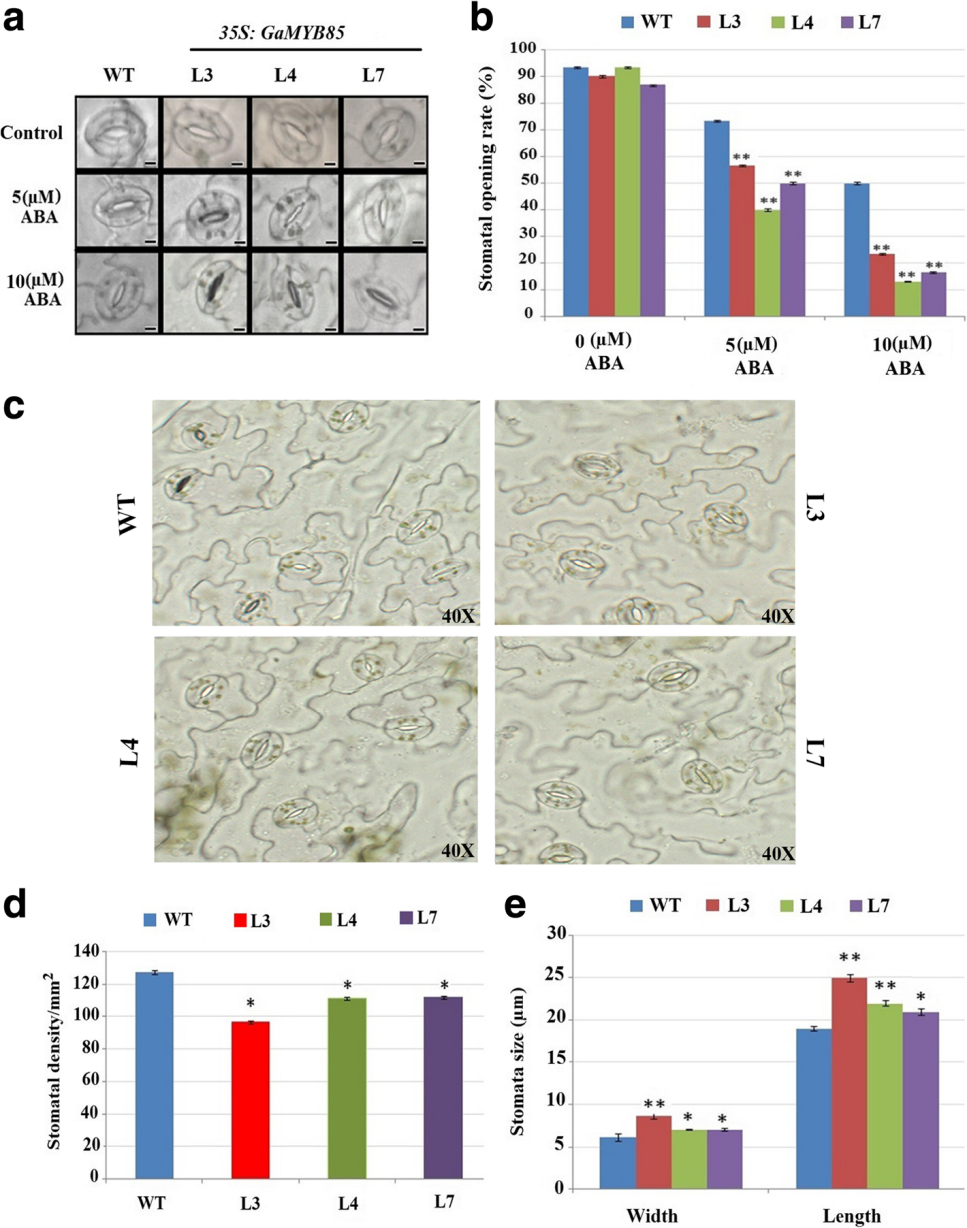
ABA induces positive stomatal modulation in *35S:GaMYB85*-overexpressing lines. **a** The stomatal pores of *35S:GaMYB85* and WT were photographed following treatment with 0, 5, and 10 μM ABA. Five views from three replicate plants were observed at ×40 magnification **b** Quantitative comparisons of the stomatal pores of *35S:GaMYB85* and WT plants following treatment with 0, 5, and 10 μM ABA. The data shown are the mean values of three replicates ± SD (*n* = 10). Scale bars: approximately 1 μm **c** Leaf stomatal density of *35S:GaMYB85* and WT plants photographed under an OLYMPUS Bx51 microscope at ×40 magnification. **d** Quantitative comparisons of *35S:GaMYB85* and WT stomatal density **e** Width and length of guard cells measured using ImageJ software. Measurements were taken from the leaves of three plants (10 stomata from five microscopic views). Data are the means ± SD. *P < 0.05; ** *P* < 0.01

ABA production under abiotic stresses is directed to increase the sensitivity of stomatal closure, and thus to gain insight into this process, we examined the effects of ABA treatment on the stomatal opening of excised leaves of *GaMYB85* plants (Fig. 8a). When different concentrations of ABA (5 μM and 10 μM) were added to a stomata opening solution, stomatal opening was reduced, as ABA induces stomatal closure. Excised leaves of *GaMYB85* lines (L3, L4, and L7) treated with 5 μM ABA showed reduced stomatal opening rates of 40%–56%, which was further reduced to approximately 13.3%–23.3% under 10 μM ABA treatments. In contrast, WT plants had markedly higher stomatal opening rates of 73% and 50% under 5 and 10 μM ABA treatments, respectively (Fig. 8b). Moreover, WT stomatal density was also notably higher, at more than 127 per mm^2^, than with 96, 111, and 112 per mm^2^ for L3, L4, and L7, respectively (Fig. 8c and d). However, stomatal size (length to width dimensions) in L3, L4, and L7 was distinctly higher at 21–25 μm by 7–8.6 μm when compared with the 19 μm by 6.05 μm for the WT, as shown in Fig. 8e. Hence, *GaMYB85* reduced stomatal density and reduced stomatal opening, resulting in rapid ABA-induced stomata closure, which subsequently minimized water loss rates and led to improved drought tolerance.

### Transcript analysis of abiotic stress-responsive marker genes

**Fig. 9:**
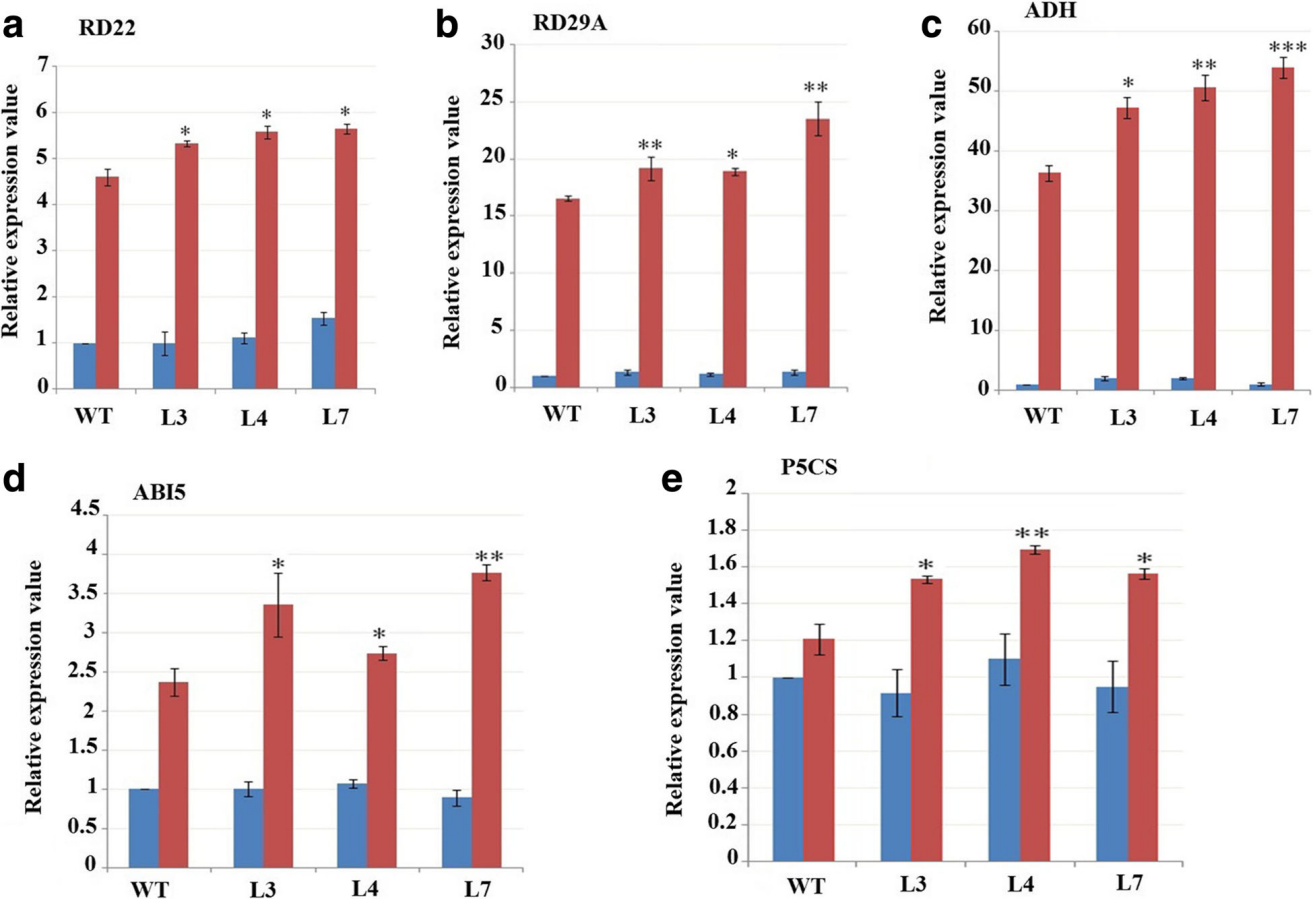
Transcript levels of ABA signaling and ABA stress-responsive marker genes in *35S: GaMYB85* and WT. (**a**–**e**) Transcript level of ABA signaling and ABA stress-responsive marker genes in two week old seedlings on (100 μM) ABA treatment for 6 h, using qRT-PCR. *AtUQB10* was used as internal control gene, with error bars of 3 biological replicates with ± SD. *P < 0.05; ** *P* < 0.01 and *** *P* < 0.001

As *Arabidopsis thaliana* plants with constitutive overexpression of *35S:GaMYB85* have ABA-dependent enhanced drought tolerance, in addition to increased stomatal closure upon ABA treatment, it would be of interest to gain a further understanding of how *GaMYB85* affects and responds to ABA stress-responsive and signaling-related genes. To this end, eight abiotic stress-responsive genes (*RD22, RD29A, RD29B, P5CS, COR15A*, *CBF, ADH,* and *Rab18*) and two ABA signaling genes (*ABI5* and *ABI3*) were selected and studied. The expression levels of five stress-responsive genes (*RD29B, COR15A, CBF, Rab18,* and *ABI3*) were nearly the same in both transgenic and WT plants in ABA-treated samples (data not shown here). However, the expression levels of five genes (*RD22, RD29A, P5CS, ADH1*, and *ABI5*) were significantly and clearly higher in L3, L4, and L7 than in the WT when treated with 100 μM ABA for 6 h (Fig. 9a-e). Thus, in response to ABA treatment, the transcript levels of ABA-induced stress marker genes may positively contribute to the improved drought stress tolerance of *GaMYB85* plants.

## Discussion

Drought stress is a worldwide dilemma that has severely affected the annual production of cotton crops. Transcription factors are well known to participate in the maintenance of plant homeostasis in response to various biotic and abiotic factors. MYB TFs, particularly R2R3 MYBs, have been widely studied in different plant species, but to date few of these genes have been functionally validated for cotton. On the basis of diploid cotton A sequencing data [35], a new candidate cloned R2R3 MYB gene, designated *GaMYB85*, was selected and ectopically expressed in *Arabidopsis thaliana,* and three overexpressing lines (L3, L4, and L7) were successfully generated and functionally validated for drought stress. The amino acid sequence of GaMYB85 was predicted to contain a conservered SANT-DBD and clusters phylogenetically with R2R3 MYB proteins from other monocots and dicots, indicating that the R2R3 MYB proteins that group together may retain an identical function based on high sequence similarity. Some R2R3 MYBs with a known role in drought response were also included in a tree analysis (Additional file 2). These share a high similarity with *GaMYB85* but cluster in different groups, which indicates that they might have a different number of SANT domains. Accordingly, these findings suggest that *GaMYB85* is a novel R2R3 MYB gene with a SANT-DBD domain.

Environmental stimuli like drought and salt stresses specifically initiate ABA production, which induces stomatal closure, contributing to strong and healthy roots and plants with efficient stomatal regulation and water-retaining abilities [38, 39]. Furthermore, drought-tolerant plants have higher endogenous proline content, reduced water loss rates, and improved relative water content, which enhance the response potential of transgenic plants against stress. Thus, to evaluate and dissect the possible molecular mechanisms and physiological roles underlying plant tolerance, we generated transgenic *Arabidopsis* that ectopically expressed *GaMYB85*. The important finding is that *35S:GaMYB85* lines have normal growth with respect to the WT, which is in contrast to previous studies in which transgenic overexpressing plants showed stunted growth and poor seed germination rates [40, 41].

In plants, turgor regulation plays an important role in the management of low water potential for normal seed germination rates under abiotic stress conditions. Thus, the observation that our *35S:GaMYB85* plants had higher germination rates when subjected to 300 mM mannitol and 150 mM NaCl treatments indicated that they have a general osmotic stress tolerance. The adverse effects of salt on plant growth can either result in osmotic stress or specific ion toxicity. The osmotic stress phase is attributable to dehydration or the presence of salt in the external solution, but specific ion toxicity is a consequence of salt accumulation in the transpiring leaves. Moreover, seeds germination rates are dependent on water movement into the seeds, whereas exposure to salinity causes leaf cells to lose water, which is attributed to reduced turgor pressure or decreased cell wall expansion [42–44]. These results are in accordance with the functions of *AtMYB102*, *AtMYB41,* and *GmMYBJ2,* which have been demonstrated to confer improved drought tolerance in response to ABA-dependent osmotic and NaCl stresses [3, 26, 30]. As elevated levels of salt can interfere with the normal molecular functioning of plants [45], *35S:GaMYB85* transgenic plants might have coped with this situation via ion homeostasis mechanisms, i.e., Na^+^ ion extrusion, Na^+^ ion compartmentalization, and Na^+^ ion reabsorption [46–48]. The roots of plants are in direct contact with the soil and more sensitive to Na^+^ ion stress, and thus provide important clues regarding normal plant growth [49]. Thus, *35S:GaMYB85* plants show a dose-dependent increase in root length growth upon salt treatment when compared with WT plants, but as transgenic plants show good fresh weight it implies that ionic stress was manifested. Furthermore, the primary root growth of seedlings was significantly decreased in WT plants growing on 200 and 300 mM mannitol MS, which clearly demonstrates the high osmotic tolerance exhibited by the *35S:GaMYB85* plants [50, 51]. As ABA hypersensitivity and insensitivity of seeds can occur during germination and post-germination stages under water deficit stress, our *35S:GaMYB85* plants were confirmed to have enhanced sensitivity to ABA both at the germinating and post-germinating stages, along with hypersensitivity to ABA inhibition of root elongation [52, 53] as shown in Fig. 3b and c. It is considered that ABA dependence might up-regulate the growth of roots and might be the major route by which *35S:GaMYB85* mediates its role in drought, salt, and cold stress signaling pathways [54, 55].

Constitutive overexpression of *35S:GaMYB85* in plants also resulted in better drought tolerance than in WT plants, which is further supported by the phenotypic and physiological changes that occur in these plants, such as decreased water loss rates and higher RWC. These results are consistent with the functional studies on *GmMYBJ2* and *TaMYB19,* which have been reported to be associated with good drought tolerance ability [30, 56]. An evaluation of water loss rates and RWC is imperative to elucidate drought avoidance mechanisms and indicates a balance between water availability and transpiration rates through the stomatal apertures of transgenic plants [57, 58]. *35S:GaMYB85* plants showed slower and lower rates of water losses with higher RWC capacity than WT plants (Fig. 6c and d), which is corroborated by recent reports on the R2R3 MYBs *GmMYBJ1* and *OsMYB48–1* [29, 59]. The total chlorophyll content of plants is linked to the physiological and transpiration efficiency of the photosynthetic machinery [60], whereas the osmoprotective molecule proline is critical for maintaining cell membrane stability, and also contributes to balancing osmotic pressure and retaining the membrane integrity of plants subjected to abiotic stresses [61]. In *35S:GaMYB85* plants, the chlorophyll and proline contents were significantly higher than those in WT plants (Fig. 6e and f), which suggests that endogenous proline levels contribute to the alleviation of water deficit stress and protect the photosynthetic apparatus from unfavorable toxic byproducts that have lethal effects on plant cells, and thereby confer drought tolerance. Interestingly, the proline levels in *35S:GaMYB85* transgenic plants are higher, even under normal condition, which suggests that this R2R3 MYB protein might specifically protect plants and confer drought tolerance via the integrated role of proline as an important osmoprotectant. Consequently, water deficit treatment indicates that drought avoidance is not only controlled by osmotic signaling but also depends on ABA-induced regulation of stomatal movement. To confirm this supposition, we examined various stomatal parameters to determine the possible roles of *35S:GaMYB85* plants in attaining good drought tolerance. In our study, *35S:GaMYB85* plants were revealed to have greater stomatal size, lower stomatal densities, and lower rates of stomatal opening than those of WT plants in response to exogenous ABA (Fig. 8b–e). These results corroborate the findings of previous studies on *35S:HDG11*-transformed tobacco, which has reduced stomatal densities but increased stomatal size [62], and are in contrast to *GbMYB5* transgenic tobacco plants, which have reduced stomatal size but the same stomatal density [25]. However, both these transgenic plants have high drought tolerance as a result of superior water retention abilities, lower transpiration rates, and reduced stomatal apertures due to elevated ABA production. Interestingly, *35S:GaMYB85* plants have reduced stomatal density but enlarged stomatal size, which can result in enhanced photosynthetic ability of plants by some unknown mechanism. This warrants further studies to gain insights into the possible function of the ABA transduction pathway in *GaMYB85*-mediated roles in reduced stomatal apertures, which helps to maintain a balance between the rates of transpiration and photosynthetic capabilities of plant during drought stress responses.

Moreover, several overexpression studies have demonstrated the use of ABA-related marker genes involved in stress-related phenotypes in *Arabidopsis* [31, 56, 63]*.* Similarly, *35S:GaMYB85* overexpression led to the up regulation of *RD22, RD29A, ADH1, AB15,* and *P5CS*. The *AB15* gene encodes a bZIP TF, and is an ABA-responsive gene known to play a prominent role during the seed germinating period during drought stress [64, 65]. Moreover, d-pyrroline-5-carboxylase synthetase (P5CS) plays a key role in the initiation step for proline synthesis during dehydration or osmotic stress, and it has been revealed that up-regulation of the *P5CS* gene in *35S:GaMYB85* plants corroborated well with the findings of studies on *OsMYB48–1*, *AtLOS5,* and *TaNAC47* [52, 53, 59]. The *RD29A* gene codes for a hydrophilic protein and is known for the presence of *DRE* or related promoter motif regions. It is activated by the combined action of DRE and ABRE *cis* motif elements, which enables it to work both in ABA-dependent and ABA-independent manners [66]. ABA-responsive expression of *RD22* is mainly due to co-expression activation of *AtMYB2/AtMYC2*. This is in contrast to *ADH1,* which only requires activated *AtMYB2* to act in a protective protein [67]. The above findings for *GaMYB85* imply that it functions in an ABA-dependent manner, and plays an important role in drought stress tolerance, which has important implications for breeding drought-tolerant cotton varieties.

## Conclusions

To summarize, ectopic expression of *GaMYB85* in *Arabidopsis* led to enhanced drought avoidance, elevated survival rates, higher accumulation of the compatible osmolyte proline, good germination rates under osmotic and salt stress, reduced water loss rates with improved RWC, increased ABA sensitivity at the germination and post-germination stages, a reduction in the number of ABA-induced stomatal apertures, and up-regulated transcription of ABA-mediated stress-responsive marker genes. In the light of the results of this study, we speculate that *35S: GaMYB85* could confer good drought, salt, and freezing tolerance in cotton crops. Our observations on the activity of the R2R3 MYB transcription factor *GaMYB85* in transgenic *Arabidopsis* plants greatly enhances our perception about its possible function in response to environmental cues, and it can be considered as a novel candidate gene for future developments in cotton breeding.

## Methods

### Transgenic plant construction and screening

The presence of the *CaMV-35S* promoter driven pCAMBIA3300-*GaMYB85* construct in *A. tumefaciens* strain *GV3101* was confirmed using a *GaMYB85* sense and antisense primer pair (Additional file 3)*,* and was ectopically expressed in *A. thaliana* (Col-0) using the floral dip method with minor modifications [68]. The infiltration media used for transformation comprised 2.215 g/L MS containing 50 g/L of sucrose, 0.5 g/L of MES, (330 μL/L) of Silwet-77 and 0.01 mg/L of 6-BA at pH 5.7. The infiltrated *Arabidopsis* was grown under the following conditions: 16 h light/8 h dark, 60% RH, and 22°C. Transgenic T_0_ seedlings were screened using 1 mg/L BASTA, which was sprayed twice on 7-d-old seedlings grown in soil pots (Additional file 4). The surviving seedlings were set to obtain T_1_ seeds, which were then confirmed for T-DNA integration in transgenic plants using a PCR Mighty Amp Genotyping Kit (Takara, China) as shown in (Fig. 2a), using the sense *35S* primer and the antisense gene primer. Wild-type (WT) plants were used as a control. Ten T_2_ lines with the correct segregation ratio (3:1) were selected and confirmed by qRT-PCR (Fig. 2b). Three T_3_ homozygous lines were selected and used for functional validation investigations (Additional file 5).

### Germination and root elongation assays

To evaluate germination in response to simulated osmotic, salt, and ABA stresses, seeds from *35S:GaMYB85* transgenic lines (L3, L4, and L7) and WT were sterilized, stratified, and sown in triplicate on half-strength MS supplemented with different mannitol concentrations (0,100, 200, and 300 mM), exogenous ABA (0, 0.3, 0.5, 1, 2, and 5 μM) and NaCl (0, 50, 100, 125, and 150 mM). The plants were scored based on non-green phenotype or dead cotyledons on the 10th day post-germination [69]. Furthermore, for seedling root length elongation assay on mannitol-, NaCl-, and ABA-supplemented MS, the methods described by [63] were followed. Three independent experiments were conducted and significant differences were determined using Student’s t-test.

### Drought and freezing tolerance assay

T_3_ homozygous transgenic line (L3, L4, and L7) and WT seeds were sterilized with 10% bleach for 10 min, rinsed with water, and stratified at 4 °C for 3 days before the seeds were transferred to MS. One-week-old seedlings were transferred from MS to pots containing a well-watered vermiculite and humus mix, and grown for 2 weeks. Thereafter, water was withheld for 2 weeks to simulate drought stress treatment. Subsequently, pots were re-watered and plant revival rates were scored after 3 d. Plants showing green healthy leaves after water replenishment were scored as surviving. The ratios of surviving to dead plants were calculated and the experiment was repeated three times [62]. For the freezing tolerance experiment, 3-week-old overexpressing transgenic lines and control WT plants were subjected to a temperature of −10°C for 3 h. Subsequently, survival rates were calculated as previously described by [52]. All experiments were repeated three times using seeds of WT plants and the three transgenic lines independently.

### Measurements of transpirational WLR and RWC

The water loss rate was determined as described by [70]. Detached leaves from 4-week-old test and control plants were immediately weighed to determine fresh weights (FW). Thereafter, at various time intervals, weights were recorded while samples were retained on a bench at 22 °C RT with a humidity level of 45% ± 5%. Fresh weights were calculated relative to plant initial weights. The relative water content of leaves was calculated using the method described by [71]. RWC percentage = (FW − DW)/(TW − DW) × 100. After determining fresh weights, the leaves were submerged in water for 4 h and turgid weights (TW) were recorded. Dry weights (DW) of leaves were recorded by drying samples at 80 °C for approximately 72 h.

### Proline and chlorophyll contents

Endogenous proline content in drought-stressed transgenic and WT plants was measured using a colorimetric PRO Kit (Jiancheng Bioengineering Institute, Nanjing, China). Three biological repeats were used, and proline absorbances at a wavelength of 520 nm were measured for determination of proline concentration.

Chlorophyll content was calculated using the formula described by [72] as follows: ((O.D 665 nm × 13.95 - O.D 649 nm × 6.88) + (O.D 649 nm × 24.96 - O.D 665 nm × 7.32))/(sample weight). The sample extraction was performed using 0.1 g of rosette leaves in 1.5 mL 95% ethanol at RT in a dark room. The absorbances of extracted chlorophyll were measured at 649 nm and 665 nm. Test and control samples were measured three times and the results were averaged. The means were compared using Duncan’s multiple range test.

### Stomatal measurements and determination of opening rate in response to abscisic acid treatment

To investigate if stomatal pore closure is sensitive to ABA, we examined the effect of ectopic expression of *GaMYB85* on stomatal opening rate of leaves under a microscope. The stomatal pores were initially opened by placing detached leaves from 3-week-old plants in a stomata opening solution (10 mM CaCl_2_, 50 mM KCl, and 5 mM MES, pH 6.15) [73] for 2 h in an illuminated growth chamber at 95% RH. Stomatal apertures were determined after 2.5 h. Two concentrations of ABA (5 and 10 μM) were applied, whereas control experiments were performed without using ABA. Samples of young leaf epidermis of transgenic and WT plants were peeled and observed under an OLYMPUS Bx51 microscope for stomata density evaluation and measurement of guard cell width to length ratio. IMAGEJ 1.51d software [74] was used to measure the size of guard cells.

### Expression analysis of marker genes by qRT-PCR

For transcript analysis of stress-responsive marker genes, 2-week-old transgenic and WT seedlings treated with 100 μM ABA were used. RNA extraction was performed using an RNA-prep Pure Plant kit (Tiangen, China), followed by first-strand cDNA synthesis using PrimeScript RT Master Mix (Takara, Clontech, China). Quantitative RT-PCR was carried out using a 7900HT detection system (Applied Biosystems), using SYBR Premix ExTaq™ (Takara, Clontech, China) according to manufacturer’s protocol. *AtUBQ10* (Accession no: AT4G05320) was used as a control gene. Three biological replicates were run using independent cDNA preparations and three technical replicates, and relative transcripts were computed using the 2^-∆∆Ct^ method based on CT values [75].

### Sequence and phylogenetic analysis

*GaMYB85* was retrieved from the cotton genome project database (http: //cgp.genomics.org.cn/page/species/blast.jsp.) and designated *GaMYB85* (cotton_A_21601). Motif analysis was performed using SMART (http://smart.embl-heidelberg.de/), whereas intron–exon analysis was carried out using the GENE Structure Display Server available online (http://gsds.cbi.pku.edu.cn). GaMYB85 protein physio-chemical properties, such as MW and pI, were evaluated using the ExPASy proteomic portal (http://www.expasy.org/proteomics). Monocot and dicot R2R3 MYB homologs were retrieved using the Blastp tool (https://www.ncbi.nlm.nih.gov/) and known R2R3 MYB were used in the construction of a cladogram based on the neighbor-joining method using MEGA 6.0. The multiple sequence alignment of *GaMYB85* R2R3 homologs was performed using the EMBL-EBI Muscle online tool (http://www.ebi.ac.uk/Tools/msa/muscle/).

### Statistical analysis of data

Statistical analysis was performed on data derived from three independent replicate experiments, using analysis of variance and Student’s t-test. Duncan’s multiple range test was applied to determine variation among treatment means of test and control lines.

## Additional files

Additional file 1: Bioinformatics sequence analysis of *GaMYB85 protein*. (DOC 660 kb)

Additional file 2: *Alignment of GaMYB85 with the deduced homologous amino acid sequences of R2R3 MYB retrieved from Blastp NCBI and known R2R3 MYB*. (DOCX 469 kb)

Additional file 3: The primers sequences used for *GaMYB85* study. (DOCX 13 kb)

Additional file 4: *35S:GaMYB85* over-expressed positive transgenic plants screening in BASTA screening at T_0_ and T_3_ stages. (DOCX 1186 kb)

Additional file 5: Survival percentage of *35S:GaMYB85* transgenic plants in 6% BASTA selection medium. (DOCX 11 kb)

## Abbreviations

ABA: Abscisic acid
DBD: DNA binding domain;
DW: Dry weight
FW: Fresh weight
*GaMYB85*: Gene in *Gossipium arboreum*;
kD: kilo Dalton
mM: millimolar
MW: Molecular weight
OD: Optical density
pI: Isoelectric point
RT: Room temperature
TF: Transcription factor
TW: Turgid weight
WT: Wild-type
μM: micromolar
