# Supplementary material for: GaMYB85, an R2R3 MYB gene, in transgenic Arabidopsis plays an important role in drought tolerance
Source: BMC Plant Biol. 2017 Aug 22;17:142. doi: 10.1186/s12870-017-1078-3 (PMC5568319; doi:10.1186/s12870-017-1078-3)

**Additional file 1** Bioinformatics sequence analysis of *GaMYB85 protein.* **a** Conserved domains analysis of *GaMYB85 showing R2R3 MYB SANT domain* **b** Predicted protein structure of *GaMYB85* by SMART online tool depict the presence of single *SANT* domain (1) 38 AA and (2) 86 *AA, SWI3, ADA2, NCoR* and *TFIIIB DBD* with (E value: 2.68e_14); 2 low complexity segments starting at 136 and end at 149 AA respectively. **c** Intron-exon sequence analysis of *GaMYB85* by (http://gsds.cbi.pku.edu.cn/). **d** Computation of *GaMYB85 protein* pI/MW by proteomics ExPASy website. **e** Secondary Structure calculation of *GaMYB85 protein* by GOR ExPASy online website.


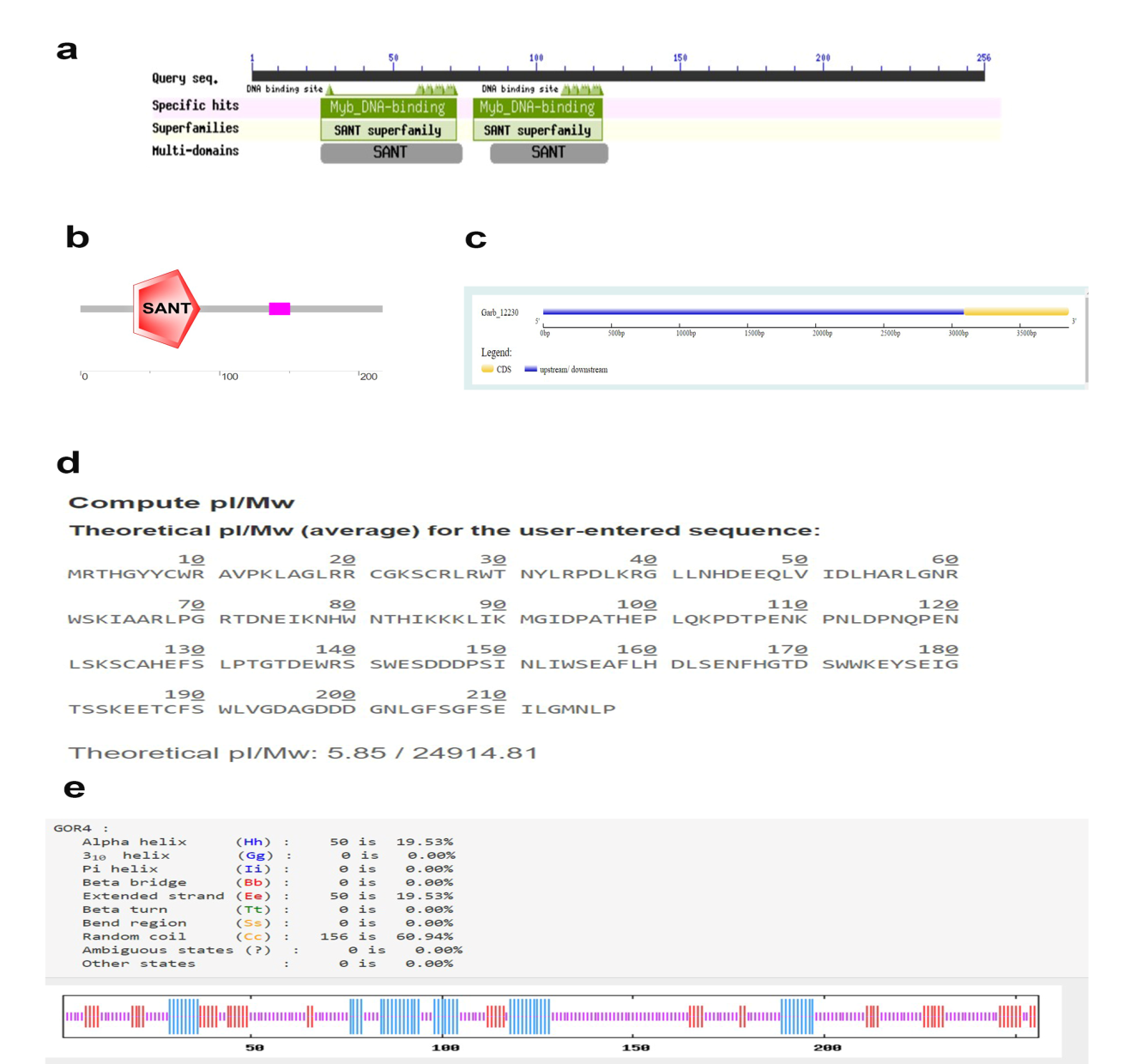

Supplement: Supplementary file 1 — Bioinformatics sequence analysis of GaMYB85 protein. (DOC 660 kb) [file 12870_2017_1078_MOESM1_ESM.doc]
