# Supplementary material for: GaMYB85, an R2R3 MYB gene, in transgenic Arabidopsis plays an important role in drought tolerance
Source: BMC Plant Biol. 2017 Aug 22;17:142. doi: 10.1186/s12870-017-1078-3 (PMC5568319; doi:10.1186/s12870-017-1078-3)

**Additional file 2:** Alignment of *GaMYB85* with the deduced homologous amino acid sequences of R2R3 MYB retrieved from Blastp NCBI and known R2R3 MYB . The gene bank accession number for *A.thaliana* :ABK28645; *A.thaliana* 85: NP567664; *N.tabacum*:XP01645541; *O.sativa*: BAT09117; *Zea mays*: NP001307849; *Vitis vinefera*: CB124555; *Theobroma caco*: XP 007038425; *G.hirsutum*: NP00131424; *Hordeum vulgarae*: BAJ98594; *GmMYBJ1*: KC7514531; OsMYB2: AK120551; *T.aestivum*: AEV91158; *TaMYB2A*: AY615199; *GbMYB5*: JF820389; *OsMYB48.1* : LoC_Os01g74410.2 ; *OsMYB48.2* : LoC_Os01g74410.1. “*” is used to indicate the consensus sequence, the “.**a**” alignment show the R2 domain , “**-b**” indicates the R3 domain of the R2R3MYB.

**a).**

**
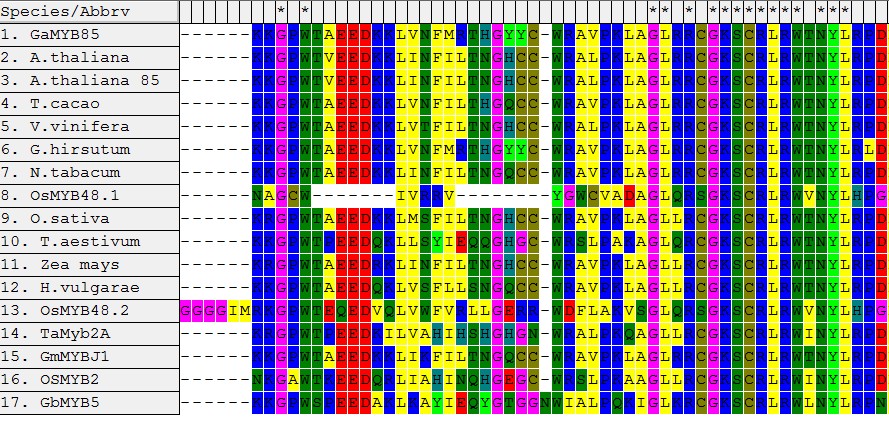
**

**b)**


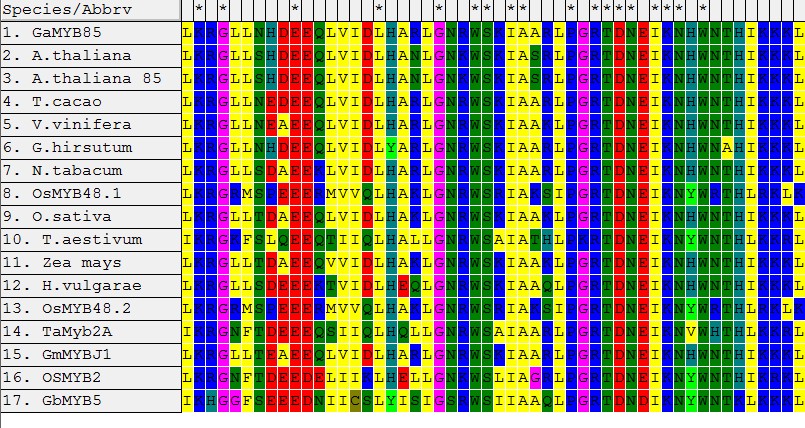

Supplement: Supplementary file 2 — Alignment of GaMYB85 with the deduced homologous amino acid sequences of R2R3 MYB retrieved from Blastp NCBI and known R2R3 MYB. (DOCX 469 kb) [file 12870_2017_1078_MOESM2_ESM.docx]
