# Supplementary material for: GaMYB85, an R2R3 MYB gene, in transgenic Arabidopsis plays an important role in drought tolerance
Source: BMC Plant Biol. 2017 Aug 22;17:142. doi: 10.1186/s12870-017-1078-3 (PMC5568319; doi:10.1186/s12870-017-1078-3)
Supplement: Supplementary file 3 — The primers sequences used for GaMYB85 study. (DOCX 13 kb) [file 12870_2017_1078_MOESM3_ESM.docx]

**Additional file 3** The primers sequences used for *GaMYB85* study

| **Primer name** | **Primer Sequence 5’to3’** | **Function** |
| --- | --- | --- |
| *GaMYB85* | F: TCTAGAATGCGCACTCACGGTTATTA  R: GAGCTCTCAAGGCAAATTCATCCCTA | For ORF amplification |
| *35S primer (F)*  *GaMYB85 (R)* | F: GACGCACAATCCCACTATCC  R: GAGCTCTCAAGGCAAATTCATCCCTA | Test of transgenic Arabidopsis T_0_ |
| *qGaMYB85* | F: CAAGATTGCCTGGAAGAACTGATAA  R: GGTGTCTGGTTTTTGTAAAGGCTCA | qRT-PCR of T2 transformant lines |
| *AtUbiquitin 10* | F: AACTTTGGTTTGTGTTTTGG  R:TCGACTTGTCATTAGAAAGAAAGAGATAA | qRT-PCR control gene |
| *ABI3* | F: CACAGCCAGAGTTCCTTCCTTTACT  R: TAGTTGCTGAGGAACACAAACGG | Marker gene for qRT-PCR |
| *ABI5* | F: AGAGGGATAGCGAACGAGTCTAGTC  R: GTTCGGGTTTGGATTAGGTTTAGG | Marker gene for qRT-PCR |
| *RD22* | F: ACTTGGTAAATATCACGTCAGGGCT  R: CTGAGGTGTTCTTGTGGCATACC | Marker gene for qRT-PCR |
| *RD29A* | \| F:GATAACGTTGGAGGAAGAGTCGG \| \| --- \|   R:TCCTGATTCACCTGGAAATTTCG | Marker gene for qRT-PCR |
| *RD29B* | F: CCGACAAGAGGTGATGTGAAAGTAG  R:GTGTAACCTAGCTTTGAGGCAACG | Marker gene for qRT-PCR |
| *P5CS* | F: GAGCAATGGAGTCACTTTGTATGG  R: TTCCTCTCATTATCCATCTCGTTG | Marker gene for qRT-PCR |
| *ADH* | F: ATGAAGCTGGAGGGATTGTTGAG  R: AGAGGAGCATCCGGATTGATCTTA | Marker gene for qRT-PCR |
| *COR15A* | F: GTGACGGATAAAACAAAAGAGG  R: GACCCTACTTTGTGGCATCCTT | Marker gene for qRT-PCR |
| *CBF* | F: GGATGCCGACTTTGTTGGATAATA  R: TAGTAACTCCAAAGCGACACGTCA | Marker gene for qRT-PCR |
| *Rab18* | F: GCAGTATGACGAGTACGGAAATCC  R: CCTTGTCCATCATCCGAGCTAGA | Marker gene for qRT-PCR |
