# Supplementary material for: GaMYB85, an R2R3 MYB gene, in transgenic Arabidopsis plays an important role in drought tolerance
Source: BMC Plant Biol. 2017 Aug 22;17:142. doi: 10.1186/s12870-017-1078-3 (PMC5568319; doi:10.1186/s12870-017-1078-3)

**Additional file 4** *35S:GaMYB85* over-expressed positive transgenic plants screening in BASTA screening at T_0_ and T_3_ stages. **a** The T_0_ *35S:GaMYB85* positive transgenic plants screening with 1% BASTA spray. The 7 day old grown seedlings were sprayed twice following 3-4 days intervals. After 3-4 days, survived green healthy grown seedling were shifted to new pots and set to get T_1_ seeds. WT was used as control in the pots without BASTA spray. BASTA selected T_3_ positives transgenic lines after one week growth on 6% BASTA supplemented media plates. WT was used as control on 6% BASTA selective medium plates.


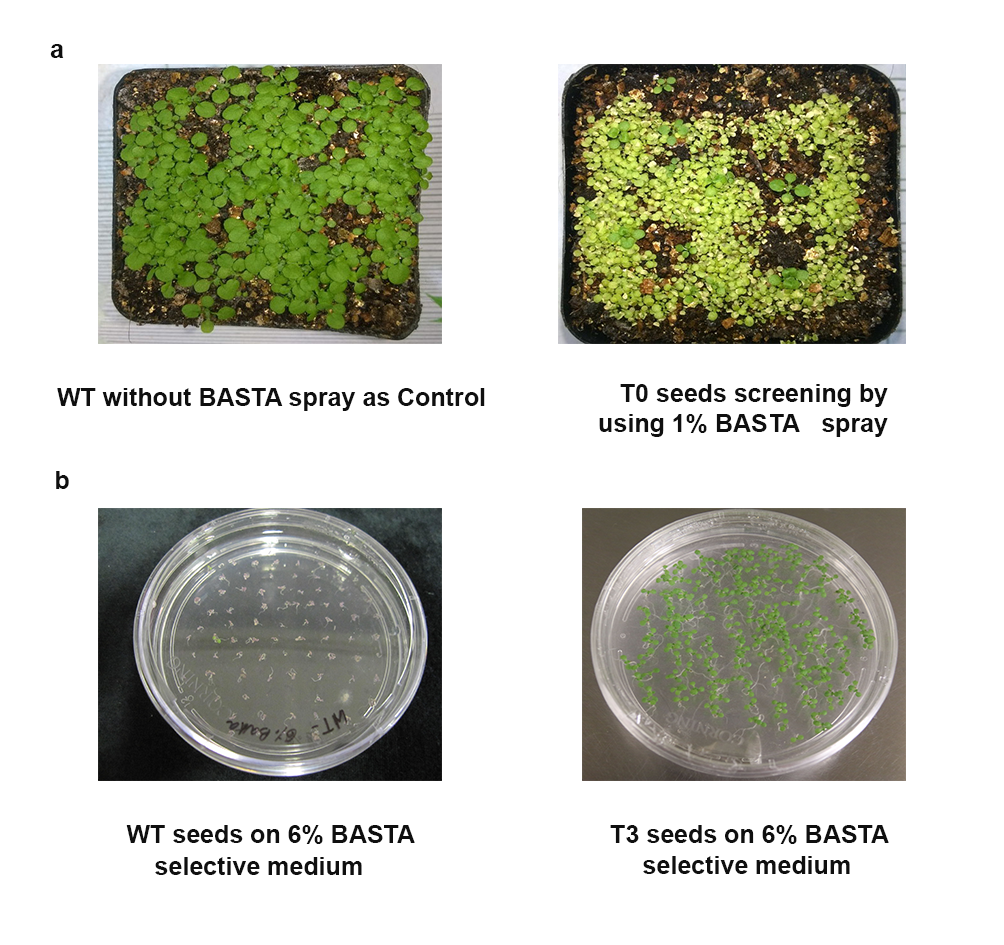

Supplement: Supplementary file 4 — 35S:GaMYB85 over-expressed positive transgenic plants screening in BASTA screening at T0 and T3 stages. (DOCX 1186 kb) [file 12870_2017_1078_MOESM4_ESM.docx]
