# Supplementary material for: GaMYB85, an R2R3 MYB gene, in transgenic Arabidopsis plays an important role in drought tolerance
Source: BMC Plant Biol. 2017 Aug 22;17:142. doi: 10.1186/s12870-017-1078-3 (PMC5568319; doi:10.1186/s12870-017-1078-3)
Supplement: Supplementary file 5 — Survival percentage of 35S:GaMYB85 transgenic plants in 6% BASTA selection medium. (DOCX 11 kb) [file 12870_2017_1078_MOESM5_ESM.docx]

**Additional file 5** Survival percentage of *35S:GaMYB85* transgenic plants in 6% BASTA selection medium. **a** *35S:GaMYB85* transgenic lines (L3, L4, L7) survival rate percentage in 6% BASTA MS plates. T_2_ segregating survival ratio of 3:1 in 6% BASTA MS medium plates, followed by T_3_ transgenic lines100 percent seed survival rate on selection medium plates.

**a.**

| **Representative Lines** | **T2 generation**  **Survival (%)** | **T2 generation**  **Dead (%)** | **T3 generation**  **Survival (%)** |
| --- | --- | --- | --- |
| 1 L3 | 78 | 22 | 100 |
| 2 L4 | 70 | 30 | 100 |
| 3 L7 | 79 | 21 | 100 |
